# Case Report: Management of a 10-Year-Old Patient Who Presented With Infective Endocarditis and Stanford Type A Aortic Dissection

**DOI:** 10.3389/fcvm.2021.816213

**Published:** 2022-01-28

**Authors:** Yanxiao Liang, Mingzhi Wan, Lijie Wang, Ni Yang, Dongyu Li

**Affiliations:** ^1^Department of Cardiac Surgery, Shengjing Hospital of China Medical University, Shenyang, China; ^2^Department of Pediatrics, Pediatric Intensive Care Unit, Shengjing Hospital of China Medical University, Shenyang, China

**Keywords:** pediatric acute aortic dissection, aortic valve replacement, infective endocarditis, aortic sinus aneurysm ruptured, Stanford type A aortic dissection

## Abstract

A 10-year-old girl presented with a chief complaint of cyclic vomiting since the last 12 h and chest pain since the last 6 h. She was diagnosed with Stanford type A aortic dissection. Intraoperatively, the aortic valve was found to be bi-lobed, and infective endocarditis associated with aortic valve perforation and rupture of the aortic sinus aneurysm, was also observed. Therefore, she underwent aortic valve replacement due to an enlarged aortic root and aortic sinus repair. The perioperative recovery was good. A large amount of bloody pericardial effusion was found in this child pre-operatively. Therefore, early surgical intervention was necessary. Acute aortic dissection rarely occurs in children. There are no clinical guidelines for the management of pediatric aortic dissection. However, if a large pericardial effusion exists, emergency surgery is necessary and effective. The treatment of the valve should be based on the actual situation. It is best to give priority to valve molding, although valve replacement is required in the majority of cases for infective endocarditis.

## Introduction

Aortic dissection (AD) mostly occurs in adults over 50 years of age, with a high fatality rate (1); it is rare in children. Based on the statistical data of 12,142 patients with AD in New York State from 1996 to 2005, no children under the age of 15 years were found, and only 45 patients under the age of 21 years were found (2). Hua et al. (3) and Shamszad et al. (4) reported that the age of onset in children was mostly 0~5 years and 15~20 years, most of them were male, and case fatality rate was 3~17%. There is no relevant report in China. Abroad, the youngest patient reported was a 1-day-old (5). Here, we report a case of successful surgical management of infective endocarditis with ruptured aortic sinus aneurysm resulting in acute AD.

This study was approved by the Institutional Review Board of Shengjing Hospital of China Medical University. The patient's parents provided written informed consent for publication of the data and associated images.

## Case Description

**Figure 1:**
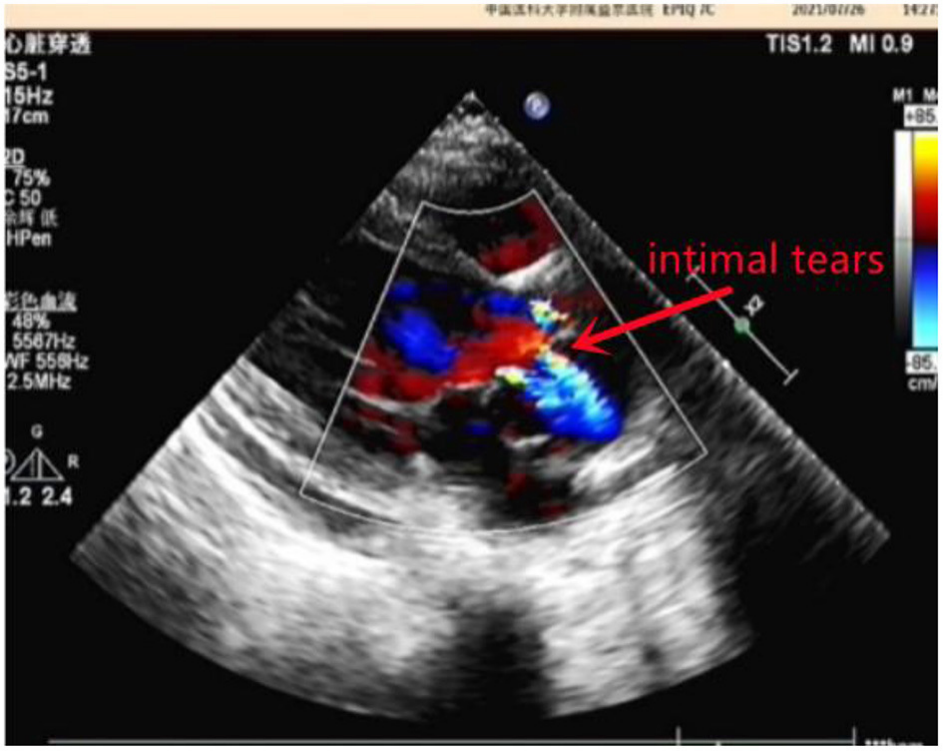
Color Doppler echocardiography.

**Figure 2:**
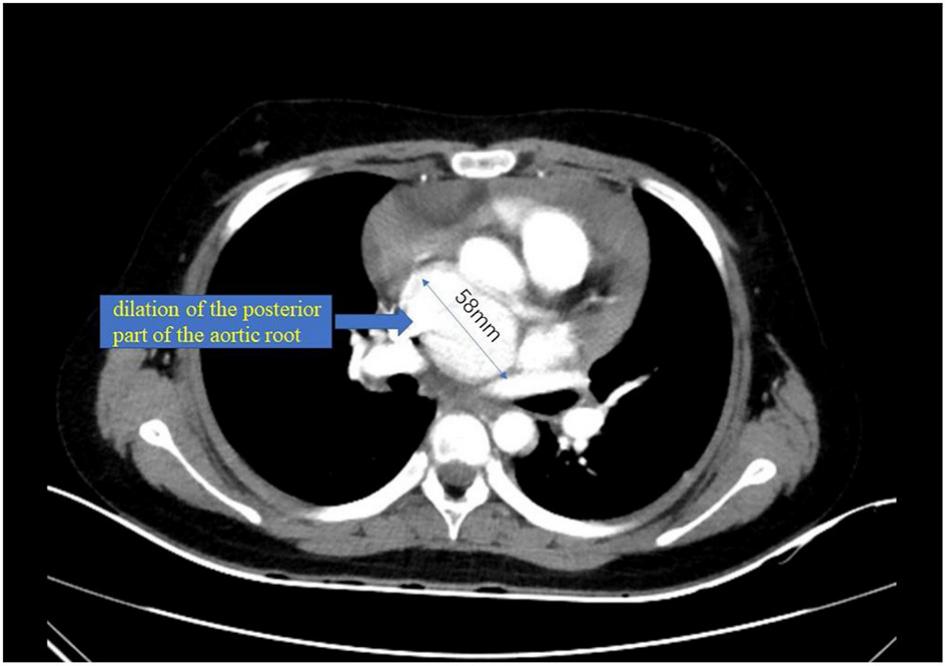
Computed tomography (CT).

**Figure 3:**
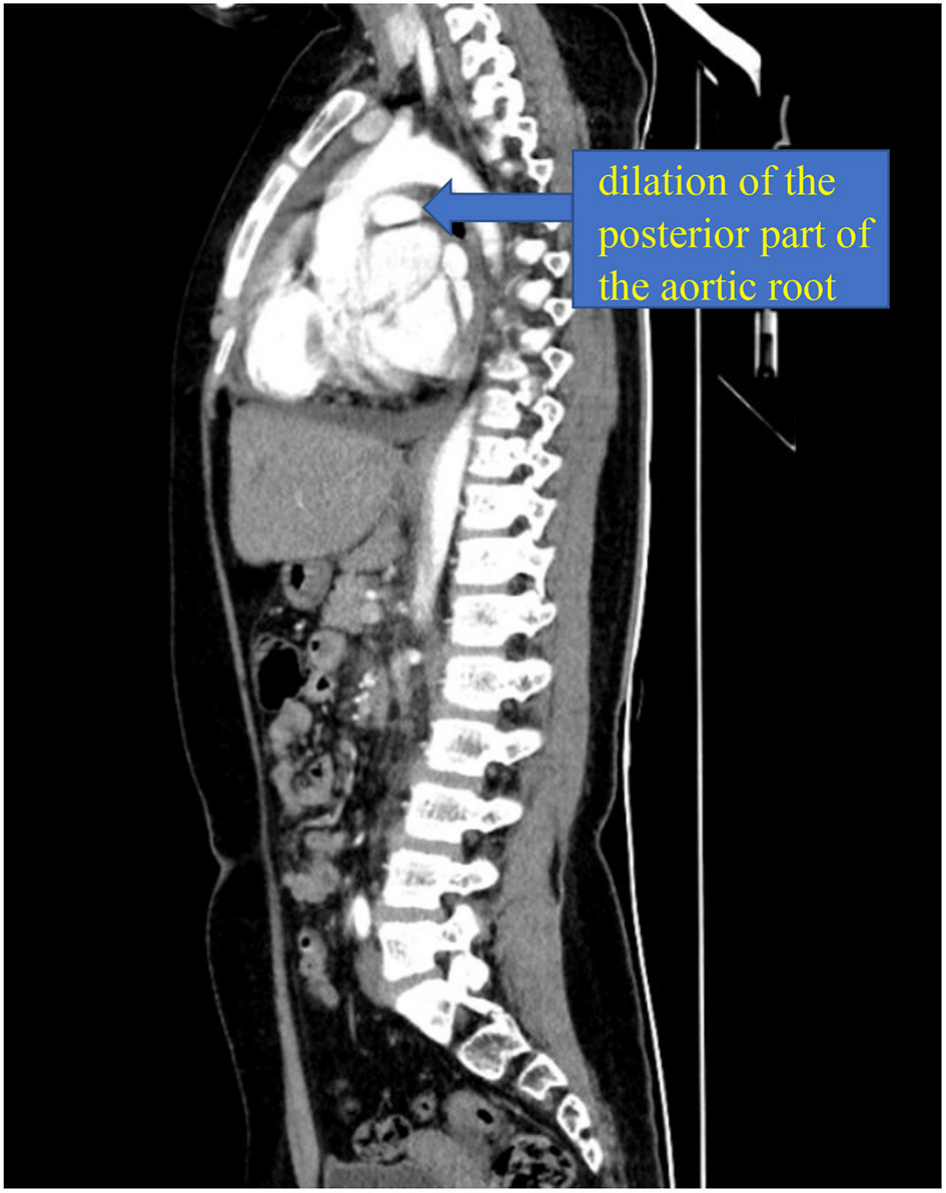
Computed tomography (CT).

**Figure 4:**
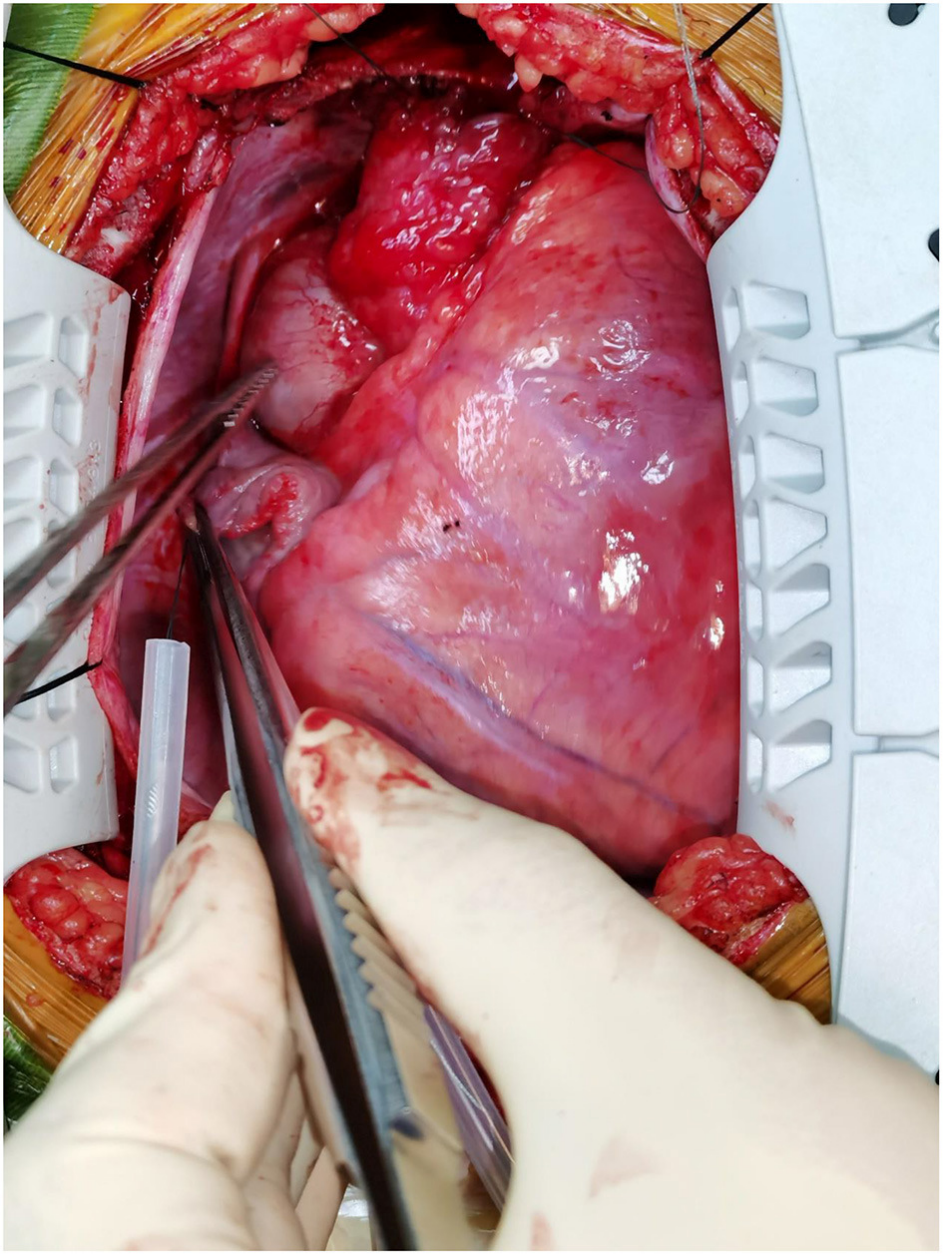
The huge aneurysm.

**Figure 5:**
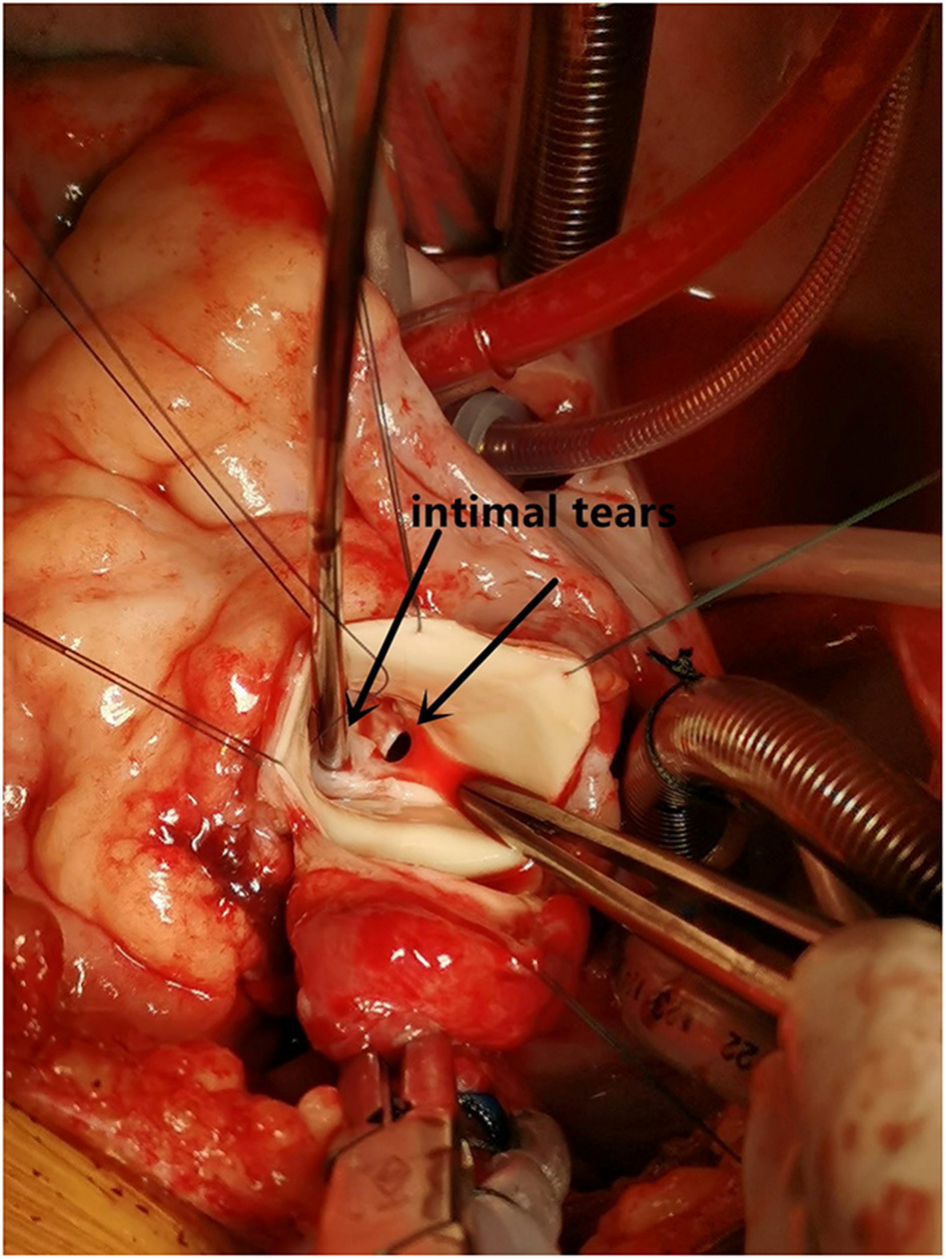
The intimal tears.

**Figure 6:**
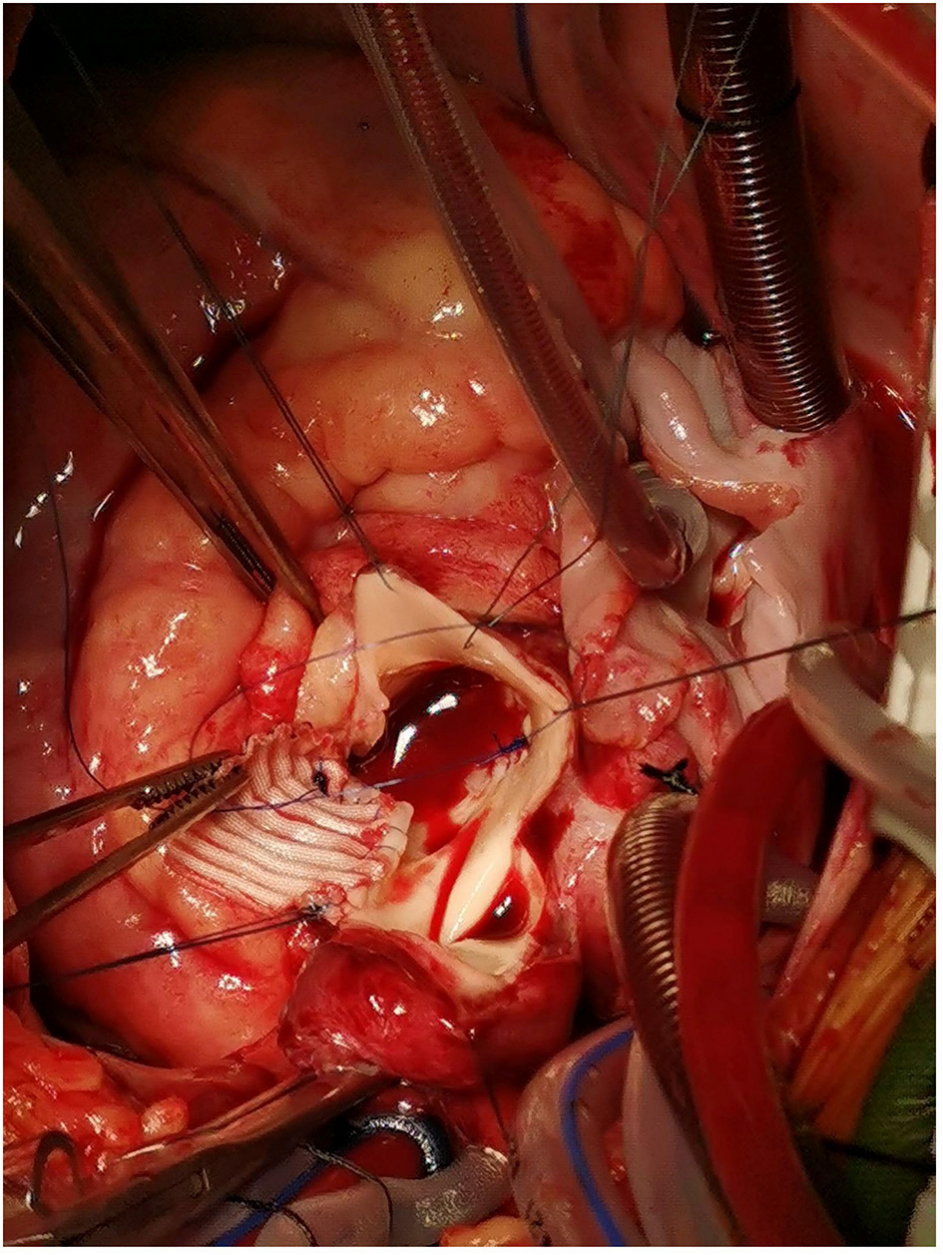
The artificial vascular patch.

A 10-year-old girl presented to our hospital with headache and nausea of 12 h duration. Physical examination revealed the following: heart rate of 120 bpm, blood pressure of 104/59 mmHg, respiratory rate of 24 breaths/min, and temperature of 36.2°C. Auxiliary examination including complete blood count revealed the following: leukocyte count, 28.85 × 10^∧^9/L; neutrophil count, 88.4%; lymphocyte count, 7.6%; hemoglobin level, 107 g/L; and platelet count, 401 × 10^∧^9/L. Troponin I level (high sensitivity) was 0.0619 ug /L, myoglobin level was 12.7 ug/L, and isoenzyme mass was 0.9 ug/L. Blood biochemistry revealed the following: total protein level, 62.1 g/L; albumin level, 34.5 g/L; alanine aminotransferase level, 26 U/L; aspartate aminotransferase level, 63 U/L; blood-glucose level, 13.83 mmol/L; creatine kinase level, 103 U/L; and creatine kinase MB isoenzyme level, 20 U/L. C-reactive protein level was 11.50 mg/L; prothrombin time, 13.8 s; prothrombin time activity, 71%; prothrombin normalized ratio, 1.3; prothrombin ratio, 1.3; activated partial thrombin time, 30 seconds; fibrinogen content, 3.1 g/L; thrombin coagulation time, 16.7 seconds; and D-dimer level, 481 ug/L. Three hours later, the patient vomited again, and began experiencing tenderness in the region of the chest anterior to the heart. Electrocardiography showed sinus tachycardia and S-T segment elevation. The patient vomited frequently, and was pale and tachypneic, with low-pitch heart sounds; her pulse pressure was increased. She was immediately transferred to the pediatric intensive care unit for further treatment, where she was sedated and given antiemetics. Physical examination revealed audible diastolic murmurs in the auscultation area of the aortic valve. On day 2, Color Doppler echocardiography revealed Stanford type A: Debakey I AD, aortic regurgitation, and massive pericardial effusion; the aortic root was widened to 50 mm in diameter (Figure 1). Computed tomography (CT) revealed a widened mediastinum, significant dilation of the posterior part of the aortic root (~58 mm at its widest point) extending up to the aortic arch. The patient had neither the exit tear, nor the involvement of the supra-aortic branches and the involvement of the abdominal aorta branches. Two lacerations could be seen above the aortic valve ring. There was blood flow through the breach from the true to the false lumen (Figures 2, 3). Consequently, on day 3, we decided to perform ascending aortic replacement. The patient was placed in the supine position, and a midsternal incision was made. Total intravenous anesthesia was administered, and cardiopulmonary bypass (in which a venous catheter was inserted into the superior and inferior vena cavae and an arterial cannula was placed in the ascending aorta) was performed. When the pericardium was opened, a large amount of hemorrhagic effusion was visible, and a huge aneurysm with a diameter of 5 × 4 cm could be seen in the posterior wall of the aortic root (Figure 4). Due to great tension, the boundaries of the aneurysm and posterior wall of the aortic root were unclear, and tissue adhesion continued upward to the junction between the ascending aorta and right pulmonary artery, causing left atrium compression. After incising the ascending aorta, bicuspid aortic valve (BAV) malformation was observed with fusion of the right and non-coronary cusp and perforation of the left coronary cusp, along with many vegetations attached to the surface. Two breaches, with diameters of 2 and 3 mm, were observed at the midpoint of the non-coronary sinus, which communicated with the aortic root aneurysm, and the surfaces of the breaches were rough with a few neoplasms visible (Figure 5). The damaged aortic wall, infected aortic valve, and neoplasms were removed with scissors, and iodophor cotton balls were used for disinfection. The aortic root was small and needed to be widened to allow for implantation of an adult-size valve. We decided to use the Konno method to enlarge the aortic valve ring. The aortic wall and valve ring were cut down along the right coronary sinus and left inferior valve lobe triangle until the ventricular septum was 1 cm from the aortic valve ring below the posterior wall of the right ventricular outflow tract and the free wall of the right ventricle. The artificial vascular patch was trimmed to an appropriate size and shaped like a tear drop. The narrowest part of the tear drop was placed on the left ventricular surface. The 4-0 line was passed through the patch, which was continuously sutured upward on the left ventricular surface and ascending aortic wall to enlarge the valve ring and ventricular septal incision. We repaired the tear in the aorta with the artificial vascular patch (Figure 6). After widening the suture, the size of the valve ring was measured and the 18# St. Jude mechanical aortic valve was used for the replacement. The valve was sutured with valve replacement suture at the normal position of the aortic valve ring, and the artificial valve was sutured with 4-0 spacer line at the position of the widened artificial vascular patch. After valve replacement, the left and right coronary openings were found to be unblocked, and the incision on the ascending aorta was closed with a 4-0-line double-layer mattress suture + continuous suture.

**Figure 7:**
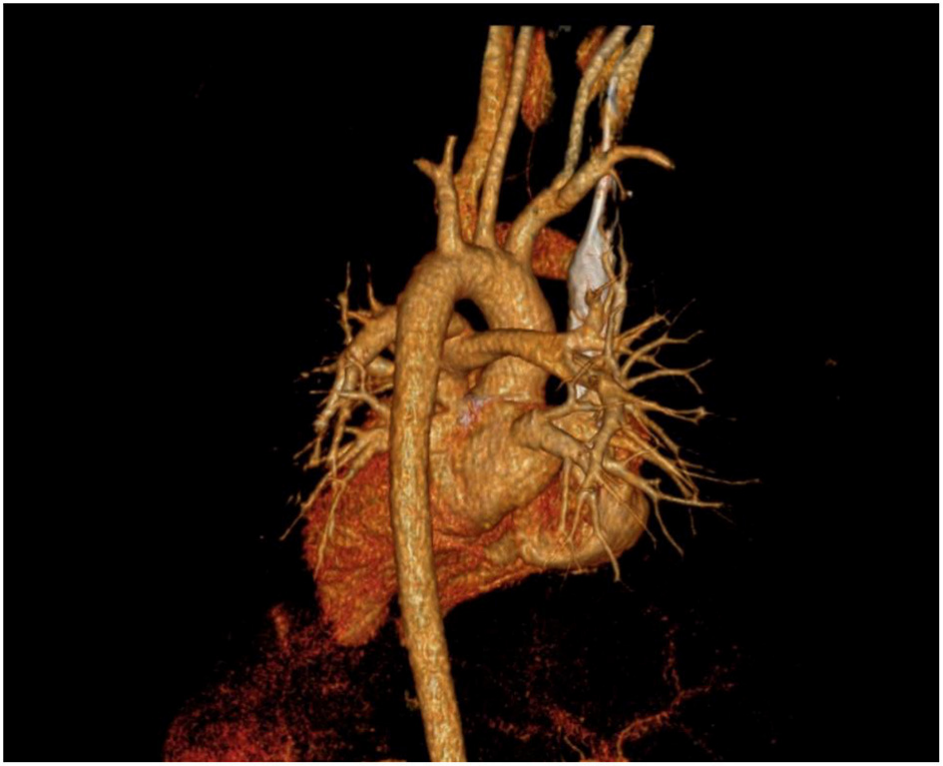
Follow-up CT.

The following day, the patient was weaned off the ventilator. She left the ICU on post-operative day 3 and was discharged from the hospital on post-operative day 10. Follow-up CT 4 weeks after surgery showed that the false lumen had disappeared (Figure 7). The patient had no symptoms such as chest pain, respiratory distress, or fever; her post-operative international normalized ratio was maintained at about 2.0, with no obvious bleeding tendency.

## Discussion

Acute AD is rare in pediatrics and can be easily misdiagnosed. A complete history and systematic physical examination are the most important parts of the assessment of children with chest pain. When the history and physical examination indicate that the pain may have a serious cause, the relevant auxiliary examinations should be urgently requested to identify the cause and determine the course of treatment. In this patient, precordium tenderness appeared 6 h after a sudden-onset headache and nausea; physical examination revealed an increase in heart rate and significant cardiomegaly. Based on the characteristics of the pain and abnormal examination findings, we considered the chest pain to be due to an organic disease and the possibility of a fatal cardiovascular disease to be high. A diagnosis of AD was made through our hospital emergency route. After the surgery, the patient's family was asked about her medical history, and they revealed that the child was hospitalized for encephalitis and septic shock 2 months ago, but no heart murmur was found at the time. After discharge, the child still had recurrent low-grade fever. It was believed that the insufficient course of anti-inflammatory treatment led to the spread of infection and colonization of the heart valve and aortic sinus by bacteria, leading to the occurrence of AD. The patient did not have hemoculture at the time. Hemocultures before and after the surgery also did not find any microbe responsible.

AD is a tear between the intima and media of the aorta due to various reasons. The intima and media of the aorta are separated and blood flows in, resulting in the formation of a true and false lumen. A typical AD shows a septum or an inner diaphragm between the true and false lumens. True and false lumens can be communicating or non-communicating. Blood can flow between the true and false lumens or form a clot (1). Hypertension and atherosclerosis are associated with 90% of adult cases of AD, while congenital and genetic diseases, such as congenital aortic stenosis, Ehlers-Danlos syndrome TYPE IV, and Marfan syndrome among others, are common in children or adolescents with AD. It can also be seen in patients with trauma, infection, and drug use.

Chest pain is a common complaint in children. Although it is mostly non-organic, a serious and life-threatening etiology may be present. The challenge for physicians is identifying the few patients with severe etiologies as early as possible. A complete history and systematic physical examination are the most important parts of the assessment in children with chest pain. When the history and physical examination indicate a potentially serious cause, auxiliary investigations should be urgently carried out to identify the etiology and initiate treatment. To the best of our knowledge, there are no clinical guidelines for the management of pediatric AD. Among conservative treatment, endovascular grafting, and valve replacement, the appropriate treatment strategy remains controversial. However, in pediatric patients, artificial blood vessels may not be the first choice due to the commercial unavailability of an appropriate graft and the problem of future growth of the artery. Therefore, in this case, we did not perform ascending aorta replacement; instead, we repaired the part of the aortic wall which was involved in the tear in the aortic intima and media. Post-operatively, the false lumen disappeared. It should be noted that this patient was suffering from BAV malformation; foreign studies have been found to be the second most common cause of heart disease in all children requiring valve replacement (6). BAV is also a major independent risk factor for AD in the Chinese population (1). When children with aortic valve disease need surgical treatment, valve repair is the first choice. If the valve cannot be repaired, or repair fails, an aortic valve replacement is compulsory. If infective endocarditis develops, mechanical valves are the best option, when available. Lifelong anticoagulant therapy is required after mechanical flap replacement. In children, the theoretical rate of anticoagulation-related complications should be higher due to lack of compliance and activity restrictions; however, the actual risk is lower in children than in adults. It has been reported in the literature that children have a 90–100% chance of avoiding thromboembolism and a 96–100% chance of avoiding bleeding during the 10 to 20-year follow-up period (7, 8). Due to age or disease, the aortic valve ring and root in children are often too small to allow for implantation of the minimum adult size (19 mm) valve. If the aortic valve ring is enlarged and the root widened at the same time, a larger prosthetic valve can be implanted and is conducive for reducing the transvalvular pressure gradient difference and improving heart function; it may also prevent the need to replace the artificial valve with a larger valve after growth and development of the child (9). In the Konno method, also known as aortic ventriculoplasty, the valve ring is fully exposed and can be expanded more effectively, while dredging the left ventricular outflow tract. However, it requires two patches, and the operation is complicated and difficult. In pediatric patients with narrower aortic annulus and root, and in some with left ventricular outflow tract stenosis, we prefer to use the Konno technique to enlarge the annulus, which allows for the implantation of larger valves. Post-operative follow-up showed that the child had a satisfactory recent effect. However, at the 3-month follow-up, the transprosthetic gradient reached 30 mmHg. Although the patient is currently asymptomatic and has good cardiac function, it is not certain whether she will require more surgery and how the cardiac function will be in the future; further observation and follow up are required

Since pediatric AD is uncommon and there are no clinical guidelines for its management, we should keep in mind that an early surgical approach with aortic replacement is sometimes necessary instead of conservative treatment or endovascular grafting.

## Data Availability Statement

The original contributions presented in the study are included in the article/supplementary material, further inquiries can be directed to the corresponding author/s.

## Ethics Statement

The studies involving human participants were reviewed and approved by the Ethics Committee of Shengjing Hospital of China Medical University. Written informed consent was obtained from the patient's family for the publication of any potentially identifiable images or data included in this article. Patient consent form was read and signed by the patient's parents.

## Author Contributions

YL participated in data collection, data analysis, and manuscript writing. MW participated in data collection and data analysis. NY participated in data analysis. LW participated in project development. DL participated in project development, data analysis, and manuscript writing. All authors read and approved the manuscript.

## Conflict of Interest

The authors declare that the research was conducted in the absence of any commercial or financial relationships that could be construed as a potential conflict of interest.

## Publisher's Note

All claims expressed in this article are solely those of the authors and do not necessarily represent those of their affiliated organizations, or those of the publisher, the editors and the reviewers. Any product that may be evaluated in this article, or claim that may be made by its manufacturer, is not guaranteed or endorsed by the publisher.
